# Global FDR control across multiple RNAseq experiments

**DOI:** 10.1093/bioinformatics/btac718

**Published:** 2022-11-03

**Authors:** Lathan Liou, Milena Hornburg, David S Robertson

**Affiliations:** Merck Research Laboratories, Merck & Co., Kenilworth, NJ 07033, USA; Merck Research Laboratories, Merck & Co., Kenilworth, NJ 07033, USA; MRC Biostatistics Unit, University of Cambridge, Cambridge CB2 0SR, UK

## Abstract

**Motivation:**

While classical approaches for controlling the false discovery rate (FDR) of RNA sequencing (RNAseq) experiments have been well described, modern research workflows and growing databases enable a new paradigm of controlling the FDR globally across RNAseq experiments in the past, present and future. The simplest analysis strategy that analyses each RNAseq experiment separately and applies an FDR correction method can lead to inflation of the overall FDR. We propose applying recently developed methodology for online multiple hypothesis testing to control the global FDR in a principled way across multiple RNAseq experiments.

**Results:**

We show that repeated application of classical repeated offline approaches has variable control of global FDR of RNAseq experiments over time. We demonstrate that the online FDR algorithms are a principled way to control FDR. Furthermore, in certain simulation scenarios, we observe empirically that online approaches have comparable power to repeated offline approaches.

**Availability and implementation:**

The onlineFDR package is freely available at http://www.bioconductor.org/packages/onlineFDR. Additional code used for the simulation studies can be found at https://github.com/latlio/onlinefdr_rnaseq_simulation.

**Supplementary information:**

Supplementary data are available at *Bioinformatics* online.

## 1 Introduction

RNA sequencing (RNAseq) is a powerful and widely used tool to profile the expression of thousands of transcripts or genes in parallel, allowing scientists to study a biological system at the transcriptome level. Most RNAseq experiments aim to compare two biological states (e.g. treated and untreated) to better understand the biological differences between the two states. At the core of the analysis of these experimental workflows is the differential expression analysis that compares the expression of genes between those two states. Since hundreds to thousands of genes are simultaneously tested in high-throughput experiments for their differential expression between two biological states, correcting for multiple hypothesis testing is essential to control the rate of type I errors (i.e. a false positive differential expression result). Earlier methods aimed to control the family-wise error rate (FWER), but this can be highly conservative—controlling the probability of *any* false positives comes at the cost of greatly reduced power to detect true positives (Korthauer *et al.*, 2019). The false discovery rate (FDR), which is the expected proportion of discoveries that are falsely rejected (Benjamini and Hochberg, 1995), was more recently proposed as an alternative less conservative metric to the FWER. FDR control has become a largely accepted standard in genomics (Chen *et al.*, 2010). Standard RNAseq analysis approaches like DESeq2 and limma typically rely on the Benjamini–Hochberg (BH) procedure to control the FDR (Love *et al.*, 2014; Ritchie *et al.*, 2015).

**Fig. 1.**
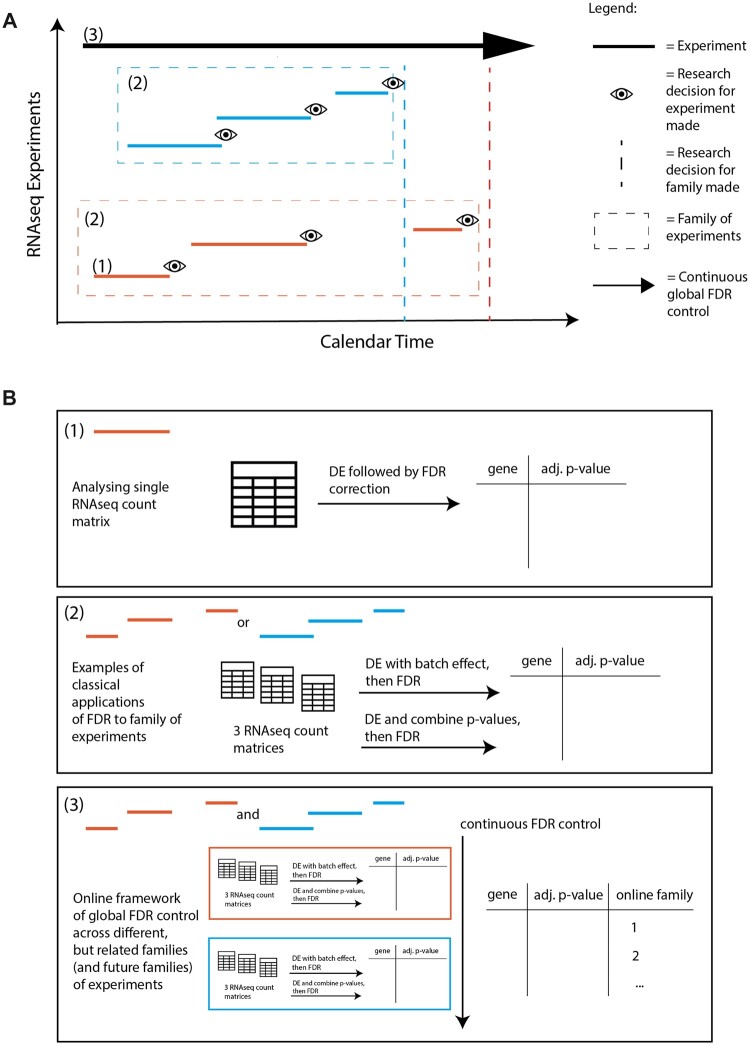
(**A**) Illustration of different settings of RNAseq experiments arriving over time. Each line represents one RNAseq experiment that includes replicate samples for two or more biological states. The different colours represent different families of experiments, as defined by distinct biological research questions. (**B**) Illustrations of different classical FDR control approaches in settings 1 and 2 and global FDR control in an online multiple hypothesis testing paradigm in setting 3

**Fig. 2.**
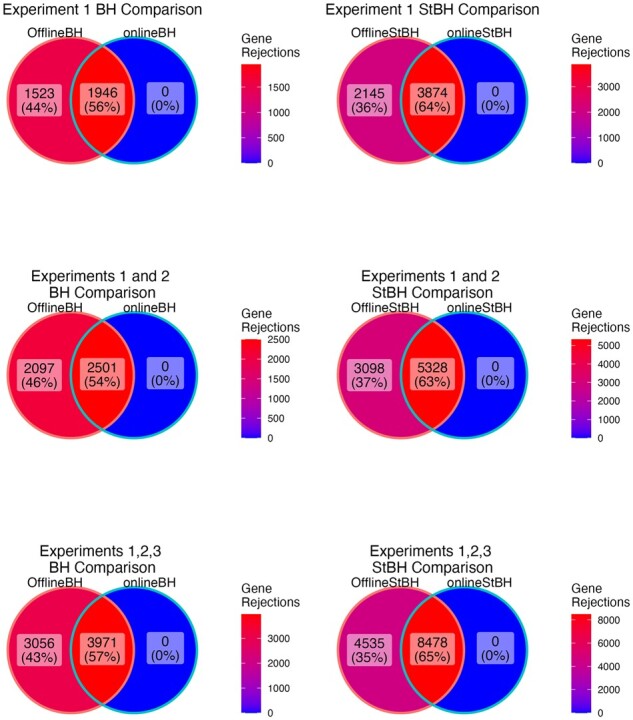
Overlap in genes declared differentially expressed between the repeated offline and online approaches. Numbers of differentially expressed genes are summed across experiments 1 and 2, and across experiments 1, 2 and 3. α=0.05

A typical RNAseq analysis involves applying a differential expression analysis method to an RNAseq count matrix, where rows are genes, columns are replicate samples for each condition and the values in the matrix are read counts, generating a gene-*p*-value matrix (Fig. 1B, setting 1). Often, an FDR correction (e.g. BH) is then applied to this gene-*p*-value matrix to generate multiplicity-adjusted *p-*values, after which a research decision may be made (Fig. 1A). However, if multiple RNAseq experiments have the same comparison groups of interest and sufficient metadata to adjust for any confounding differences in experimental or study design, they may be combined in some way to pool information. We refer to this as a *family of experiments*. That is, similar experiments performed over calendar time, which could include different investigators, labs, machines (as opposed to time-course experiments). In this setting, there are several classical analysis strategies that are typically performed (Fig. 2A). One approach combines multiple gene-*p-*value matrices into a larger one, adjusting for batch effect, and performs differential expression analysis with an FDR correction. Another performs differential expression independently on each gene-*p-*value matrix, combines *p-*values for each gene, and then performs an FDR correction on the combined *p-*values.

As is increasingly common in many pharmaceutical target discovery programs, many compounds are often tested over time, so we argue that there is a rationale to globally control the FDR across multiple families of RNAseq experiments, going beyond FDR corrections of individual RNAseq experiments or a single family of RNAseq experiments. As an example, suppose that there are 10 families of RNAseq experiments that have different, but related, treatment conditions (e.g. transcriptome of the same biological samples but treated with different anti-inflammatory compounds). These families of experiments were performed at different times and perhaps at different labs and have different treatment conditions. The FDR has been controlled within each family, using the classical approaches as described above, but the *global* FDR across families may be inflated from separately applying an FDR correction within each family.

A solution to this problem is to apply recently developed methodology for *online* multiple hypothesis testing to analyse multiple families of RNAseq experiments over time in a principled way, guaranteeing global FDR control (Fig. 1C). In the framework of online multiple hypothesis testing, hypotheses arrive over time to be tested and the investigator must decide whether to reject the current null hypothesis(es) without knowing the future *p*-values (or even the total number of hypotheses to be tested), but only knowing the historic decisions to date.

In practice, Robertson *et al.* (2019) describe scenarios in which online multiple hypothesis testing can be applied to growing public biological databases such as the International Mouse Phenotype Consortium. In addition, open-source repositories of high-throughput gene expression data such as Gene Expression Omnibus are examples of growing databases where similar RNAseq experiments are collected over time (Edgar *et al.*, 2002).

In the context of multiple families of RNAseq experiments, the online methodologies have two important characteristics. First, using our hypothetical example above, any hypothesis rejections that were made on genes following treatment with anti-inflammatory drug X will not change with the addition of RNAseq differential expression results from anti-inflammatory drug Y. Second, online methods can accommodate future data that arrives over time. The most compelling argument as to why historic decisions should not change is that there is no practical course of action. For instance, if additional assays or experiments within a research program were already decided based on the RNAseq experiments of anti-inflammatory drug X, which cannot change. However, the online methodology uses information gained from previous hypothesis tests to inform how large a significance threshold future hypothesis testing can use.

In this article, we compare the intuitive approach of separately applying FDR corrections to each family of experiments (termed ‘offline’ testing) with online multiple hypothesis testing, using both real-world RNAseq experiments as a motivating example and simulated RNAseq data to explore broad patterns in online and offline behaviours with respect to FDR and power. To the best of our knowledge, our study is the first to apply online multiple testing methods to RNAseq data.

In Table 1, we provide a glossary of the terminology used in the article.

**Table 1.** Glossary table

Term	Definition
Experiment	RNAseq experiment that generates a single count matrix (rows are genes, columns are samples). A subsequent classical differential expression analysis that outputs a gene-*p*-value matrix can be used to answer a biological research question.
Family of experiments	Multiple RNAseq experiments that have been conducted by different investigators, labs or timepoints that have the same comparison groups of interest and sufficient metadata to adjust for any confounding differences in experimental or study design, and so may be combined to give a single gene-*p*-value matrix.
Gene-*p*-value matrix	Output of classical differential expression analysis, i.e. a dataframe of *p*-values for each gene.
Rejected hypothesis	Gene declared differentially expressed.
Incorrectly rejected hypothesis	Gene incorrectly declared differentially expressed.
Uncorrected	No FDR adjustment applied, so that a gene is declared differentially expressed if the corresponding *p-*value is less than *α*.
Offline	Separate application of an FDR correction method, such as BH, to each gene-*p*-value matrix or each classically pooled gene-*p*-value matrix from a *single* family of experiments.
Online	Application of online multiple hypothesis testing algorithms (e.g. onlineBH, onlineStBH, or onlinePRDS) across multiple gene-*p*-value matrices from *multiple* families of experiments.

## 2 Materials and methods

### 2.1 FDR control paradigms

We use the terminology of ‘offline’ approaches to refer to taking as input a single gene-*p*-value matrix and output a set of rejection decisions for all hypotheses at once to control the FDR. Offline approaches assume no knowledge of previous or future data and only considers the *p-*values obtained at any given time. This approach is what is widely performed in academia and industry; for instance applying a BH correction once on an RNAseq gene-*p-*value matrix.

In our article, we consider the implications of applying a repeated offline approach, applying an FDR correction separately to each gene-*p*-value matrix as they arrive over time, ignoring the fact that multiple experiments are being analysed over time. For repeated offline methods, we use the BH procedure (Benjamini and Hochberg, 1995) and Storey-BH (StBH) procedure (Storey, 2002). Given *p*-values $p_{1},p_{2},\ldots ,p_{N}$ and a significance level *α*, the BH procedure is as follows:

1. Order the *p-*values from smallest to largest, giving ordered *p-*values $p_{\left(1\right)}\leq \cdots \leq p_{\left(N\right)}$ and define $p_{\left(0\right)}=0$;
2. Let $i^{*}$ be the maximal index such that $p_{\left(i^{*}\right)}\leq i\alpha /N$;
3. Reject null hypothesis *H_j_* for every test with $p_{j}\leq p_{\left(i^{*}\right)}$.

StBH improves upon the original BH procedure by letting users choose a parameter λ∈(0,1), which is used to estimate the proportion of nulls in the *p-*value set, defined as:

$$\frac{1+\Sigma_{i=1}^{N}1\{p_{i}>\lambda \}}{N(1-\lambda )}$$

The motivation for using StBH is that BH might be overly conservative when there are many expected rejections with a strong signal, so StBH is more adaptive to the set of *p-*values at hand, which will provide important intuition to the online analogues of these algorithms. It is important to note that BH relies on the assumption that the *p-*values are positively dependent (Benjamini and Yekutieli, 2001) and StBH requires independent *p*-values (Storey, 2002) for provable FDR control.

In contrast, the online approach allows for informed hypothesis-driven decision-making using prior information and offers guaranteed FDR control for past, present and potential future families of experiments. With repeated offline methods, there are distinct points in time when an FDR control method is applied, but with online methods, the application is continuous throughout the timeline of families of experiments: past, present and future (Fig. 1).

In the online paradigm for testing multiple families of RNAseq experiments, at each time point t∈ℕ, a gene-*p-*value matrix of *N_t_* genes arrives, corresponding to the results from a single family of experiments. In the online paradigm, no information about future families of experiments needs to be available, not even the total number of families to be tested. Each gene has an associated *p*-value and we let $P_{t}$ denote the set of *p-*values corresponding to the *t-*th gene-*p-*value matrix, given by $P_{t}:=\{P_{t,1},\ldots ,P_{t,N_{t}}\}$, where $P_{t,j}$ is the *j-*th *p-*value in gene-*p-*value matrix *t*. Here, $P_{t,j}$ is used to test the null hypothesis that the corresponding gene is not differentially expressed.

The FDR *up to the time t* is then the expected false discovery proportion (FDP) up to time *t*, i.e. the expected proportion of false discoveries up to time *t* among the rejected hypotheses (e.g. genes declared differentially expressed) up to time *t*. Letting *V*(*s*) denote the number of incorrectly rejected hypotheses and *R*(*s*) denote the number of rejected hypotheses in gene-*p-*value matrix *s*, the FDR up to time *t* is defined as follows:

$$\text{FDR}(t)\equiv E[\text{FDP}(t)]\equiv E\left(\frac{\sum_{s=1}^{t}V(s)}{\max(\sum_{s=1}^{t}R(s),1)}\right).$$

To leverage the fact that multiple *p*-values arrive at once (corresponding to a gene-*p*-value matrix for a single family of experiments becoming available), we can use so-called ‘Batch’ procedures for online control FDR as proposed by Zrnic *et al.* (2020). In brief, online Batch procedures (henceforth referred to simply as ‘online’) aim to interpolate between the offline and online settings and achieve high power, while guaranteeing FDR control at global level *α* at all times t∈ℕ. These algorithms adaptively determine a *test level α_t_* based on information about past hypothesis tests, so that the set of *p*-values $P_{t}$ are then tested using an offline algorithm (e.g. BH) at level *α_t_*. The algorithms require the prior specification of a sequence ${\left\{\gamma_{s}\right\}}_{s=1}^{\infty }$, the details of which can be found in Zrnic *et al.* (2020).

We use three online procedures: onlineBH, onlineStBH and onlinePRDS. onlineBH and onlineStBH are the online analogues of the BH procedure and Storey-BH procedures, respectively. Both onlineBH and onlineStBH require that *p*-values are independent for provable FDR control, an issue that we will return to in the Section 4. onlineStBH aims to be more adaptive to the data than onlineBH, given its requirement for a user-chosen constant λ∈(0,1) like for the Storey-BH procedure. onlinePRDS is a modified version of onlineBH that controls the FDR when the *p-*values in a single gene-*p-*value matrix are positively dependent yet independent across different gene-*p-*value matrices. Further details about these algorithms can be found in the original paper (Zrnic *et al.*, 2020). These procedures are all implemented in the R package *onlineFDR* (Robertson *et al.*, 2019), which is freely available through Bioconductor at http://www.bioconductor.org/packages/onlineFDR.

The onlineBH procedure is defined as follows:

Algorithm 1: onlineBH Algorithm

**Data:** Global FDR level *α*, non-negative sequence ${\left\{\gamma_{s}\right\}}_{s=1}^{\infty }$ such that $\sum_{s=1}^{\infty }\gamma_{s}=1$.

Set $\alpha_{1}=\gamma_{1}\alpha$;

**for**t=1,2,…**do**

  Run the BH method at level *α_t_* on gene-*p-*value matrix $P_{t}$;

Set $\alpha_{t+1}=\left(\sum_{s\leq t+1}\gamma_{s}\alpha -\sum_{s\leq t}\alpha_{s}\frac{R_{s}^{+}}{R_{s}^{+}+\sum_{r\leq t,r\neq s}R_{r}}\right)\frac{N_{t+1}+\sum_{s\leq t}R_{s}}{N_{t+1}}$;

**end**

where *R_s_* is the total number of rejections for gene-*p-*value matrix *s* and $R_{s}^{+}$ is the maximum number of rejections for gene-*p-*value matrix *s* if one *p*-value in matrix *s* is set equal to zero and all other *p*-values are held fixed (where the maximum is taken over the choice of the *p*-value which is set to zero).

The onlineStBH procedure is similar to the onlineBH procedure with an additional adaptive constant *k_s_* [which is defined in Zrnic *et al.* (2020)]:

Algorithm 2: onlineStBH Algorithm

**Data**: Global FDR level *α*, non-negative sequence ${\left\{\gamma_{s}\right\}}_{s=1}^{\infty }$ such that $\sum_{s=1}^{\infty }\gamma_{s}=1$.

Set $\alpha_{1}=\gamma_{1}\alpha$;

**for** t=1,2,… **do**

  Run the Storey-BH method at level *α_t_* on gene-*p-*value matrix $P_{t}$;

Set $\alpha_{t+1}=\left(\sum_{s\leq t+1}\gamma_{s}\alpha -\sum_{s\leq t}k_{s}\alpha_{s}\frac{R_{s}^{+}}{R_{s}^{+}+\sum_{r\leq t,r\neq s}R_{r}}\right)\frac{N_{t+1}+\sum_{s\leq t}R_{s}}{N_{t+1}}$;

**end**

Finally, the onlinePRDS procedure is defined as follows:

Algorithm 3: onlinePRDS Algorithm

**Data**: Global FDR level *α*, non-negative sequence ${\left\{\gamma_{s}\right\}}_{s=1}^{\infty }$ such that $\sum_{s=1}^{\infty }\gamma_{s}=1$.

Set $\alpha_{1}=\gamma_{1}\alpha$;

**for**t=1,2,…**do**

  Run the BH method at level *α_t_* on gene-*p-*value matrix $P_{t}$;

  Set $\alpha_{t+1}=\alpha \frac{\gamma_{t+1}}{N_{t+1}}\left(N_{t+1}+\sum_{s=1}^{t}R_{s}\right)$;

**end**

In summary, the online procedures ensure global FDR control, across all families of experiments at all times, at level *α*. This is achieved through the specification of adjusted testing levels *α_t_* so that the *p*-values in the *t*-th gene-*p*-value matrix are tested using BH (or Storey-BH) at level *α_t_*. In contrast, the repeated offline approaches to FDR control will test the *p*-values in each gene-*p*-value matrix using BH (or Storey-BH) at level *α*, which does not guarantee global FDR control at level *α* (see the simulation results in the following section).

## 3 Results

### 3.1 Real-world data application

For the online approaches, we use the real-world data to construct a hypothetical use case for online algorithms. For the purposes of illustration, we consider each experiment here as its own family. While it is possible to use the classical approaches as described in the Section 1 to pool the real-world gene-*p-*value matrices we use in this study, we instead aim to illustrate the application of online FDR control methodologies on data with real-world variability and *p-*value dependencies. We also carry out a sensitivity analysis (see below) which corresponds to the case of using online algorithms across distinct families of hypotheses.

We used three real-world RNAseq experiments procured from internal studies of Merck & Co., Inc., Kenilworth, NJ, USA, anonymized as Experiments 1, 2 and 3. All three RNAseq experiments used CT26 syngeneic colon carcinoma mouse models treated with murinized rat anti-mouse programmed cell death protein-1 (PD-1) antibody (muDX400) or vehicle control with the same experimental design such as treatment duration and dose. The three experiments were originally conducted to answer slightly different research questions and included additional samples, but we used the muDX400 and vehicle control conditions in this analysis which was present in all experiments. The order of experiments was determined by the chronological order that they were performed at Merck & Co., Inc., Kenilworth, NJ, USA. We selected vehicle control samples as our control arm and muDX400 samples as our treatment arm in a two-sample differential expression analysis. Experiment 1 contained 10 control and 10 treatment samples. Experiment 2 contained 10 control and 5 treatment samples. Experiment 3 contained 19 control and 18 treatment samples.

After filtering out ambiguously mapped genes and genes with low counts across samples using the *edgeR* package (Chen *et al.*, 2016), the count matrices from each of these experiments was voom-transformed and DE analysis was performed on each using the *limma-voom* workflow (Law *et al.*, 2014; Ritchie *et al.*, 2015). 14 708, 12 230 and 14 948 genes went into the differential expression analysis for Experiments 1, 2 and 3, respectively. We converted mouse gene symbol names to human gene symbol names using the getLDS() function with the December 2021 conversion list of the *biomaRt* package in order to provide more direct comparisons with the existing literature on anti-PD-1 gene expression profiles (Durinck *et al.*, 2009). For the repeated offline approaches, we apply offline BH and StBH corrections separately to each of the three output gene-*p-*value matrix. We acknowledge an important limitation of this use case for the online approaches, since a proportion of the same genes is tested across multiple experiments, which does not strictly fit into the online multiple hypothesis testing framework described in Section 1 (where the hypotheses are assumed to be distinct). However, we show this comparison of the online and offline FDR approaches using real-world data to highlight patterns in the number of rejections agnostic to the biological context of the hypotheses.

We explored whether there is face validity of online methods by identifying whether a previously described gene expression profile following anti-PD-1 therapy can be recapitulated (Supplementary Appendix). In particular, 18 inflammatory genes related to antigen presentation, chemokine expression, cytolytic activity and adaptive immune resistance, including CCL5, CD27, CD274 (PD-L1), CD276 (B7-H3), CD8A, CMKLR1, CXCL9, CXCR6, HLA-DQA1, HLA-DRB1, HLA-E, IDO1, LAG3, NKG7, PDCD1LG2 (PDL2), PSMB10, STAT1 and TIGIT (Cristescu *et al.*, 2018).

As a benchmark, almost all of the FDR control methods are more conservative than the uncorrected approach (defined as rejecting a hypothesis if the unadjusted *p-*value is less than *α*) at α=0.05 at all time points (Table 2). The repeated offline StBH approach make more rejections compared to repeated offline BH. This is expected because StBH is more adaptive to the data at hand and has been shown empirically to yield higher power (Storey, 2002). Zrnic *et al.* (2020) have shown theoretical guarantees that FDR will be controlled across all *p-*values, assuming *p*-values are independent. Although onlineBH and onlineStBH make fewer rejections than their offline analogues, this is to be expected in order to maintain overall FDR control. It is worth noting that in this real-world example, onlineStBH declared more genes as differentially expressed (DE) than repeated offline BH at each time point (Table 2).

**Table 2.** Cumulative number of rejections (out of total genes tested) in real-world RNAseq data across experiments

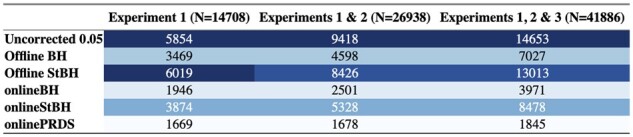

*Note*: *N* is the total number of genes tested. Blue colour scales with the number of null hypothesis rejections with dark blue for high number of rejections, light blue for low number of rejections.

The onlineBH procedure made the same rejections 56% of the time as its repeated offline analogue across the three gene-*p-*value matrices (Fig. 2). onlineStBH achieved a higher proportion of overlapping rejections of 63% across the three gene-*p-*value matrices. Online approaches are able to detect a moderately high percentage of the same DE genes as the repeated offline approaches. Although the online approaches in this real-world data example are more conservative than their corresponding offline counterparts, the repeated offline approaches do not have the long-run FDR control guarantees of the online approach. In the sensitivity analysis described below, we observed that online StBH was able to recapitulate many of the same genes that were described by Cristescu *et al.* (2018) in each of the three experiments, such as CD27, CD274, CD8A, CMKLR1, CXCL9, CXCR6, HLA-E, IDO1, LAG3, NKG7, PDCD1LG2, PSMB10, STAT1 and TIGIT (Supplementary Appendix).

Since a proportion of the same genes were measured across all three experiments, we also performed a sensitivity analysis to illustrate the case of extending FDR control across different, but related families of hypotheses (genes) (Fig. 1B). We filter each of the experiments to remove any overlapping genes in common post-differential expression analysis leaving 2634, 156 and 2874 unique genes in Experiments 1, 2, and 3, respectively. We then applied both offline and online FDR control methods to the filtered sets of genes as described above. Figures showing the overlap between the rejections made by the different procedures are given in the Supplementary Appendix.

Within the filtered genes, we see a similar pattern where the online methods are more conservative than their offline analogues, but onlineStBH makes a comparable number of rejections to offline BH (Table 3). Because the main 18 genes highlighted in the gene expression were filtered out, to identify whether biologically meaningful genes were identified, we used the enrichR tool to summarize gene set information using KEGG 2021 pathways (Chen *et al.*, 2013). Using online StBH, we identified the B-cell receptor signalling pathway as the most highly enriched pathway in filtered experiments 1 and 3. This corroborates previously demonstrated evidence that blockade of PD-1 increases B-cell activation (Thibult *et al.*, 2013).

**Table 3.** Cumulative number of rejections (out of filtered genes unique to each experiment) in real-world RNAseq data across experiments

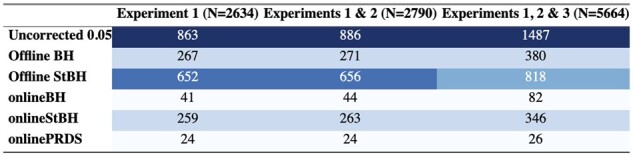

*Note*: *N* is the number of filtered genes tested. Blue colour scales with the number of null hypothesis rejections with dark blue for high number of rejections, light blue for low number of rejections.

**Table 4.** Ordered setup of number and proportion of super matrices and regular matrices

Family	Super matrix	Super matrix *π*	Regular matrix	Regular matrix *π*	Overall *π*
50	10	0.3	40	0.050	0.1
50	10	0.3	40	0.175	0.2
50	10	0.5	40	0.250	0.3
50	10	0.5	40	0.375	0.4
50	10	0.5	40	0.50	0.5

### 3.2 Simulation study

In the real-world data application, only three experiments were analysed. To explore the application of the online FDR control methods on a much larger scale, we performed an RNAseq simulation study using the *compcodeR* package (Soneson, 2014), which simulates read counts from a negative binomial distribution (see Supplementary Appendix for more details). We used one of the standard practice workflows for RNAseq differential expression analysis and applied the *voom* method (Law *et al.*, 2014) from the *limma* package (v3.50.0) (Ritchie *et al.*, 2015).

In all our simulation experiments, 50 RNAseq count matrices each with 10 000 genes were simulated to represent 50 families of experiments arriving over time (Fig. 1B, setting 3). As a sensitivity analysis, a smaller number of families (25 RNAseq count matrices) were also simulated (Supplementary Fig. S3 in the Supplementary Appendix). We emphasize again that, for the purposes of the simulations, each count matrix is considered as its own family. For each simulation run, we simulated a range of proportions of DE genes:

{0.01,0.02,…,0.09,0.1,0.2,0.3,0.4,0.5}

We focus on two-group comparisons only. We specified 5 biological replicates for both the control and experimental groups, a log-fold change simulation parameter of 1.5, a sequencing depth of 10^7^, minimum and maximum factors that are multiplied with sequencing depth of 0.9 and 1.1, respectively, and a proportion of up-regulated genes set to 0.5, which were all mostly default values in the *compcodeR* package. We then ran *limma-voom* as our differential expression analysis method. This simulation setup was replicated 1000 times independently.

In each simulation run, we applied offline BH, offline StBH and the online procedures onlineBH, onlineStBH and onlinePRDS using default parameter values as described in the Supplementary Appendix. The FDP was calculated as the number of false positives divided by the total number of genes declared DE. The average FDP across 1000 simulation runs provides an empirical approximation of the true FDR. Power was calculated as the number of truly DE genes (true positives) divided by the total number of genes declared DE (all positives) and averaged over 1000 simulation runs. For offline BH and StBH, this entailed applying BH and StBH to each of the 50 count matrices, and then calculating FDR and power.

**Fig. 3.**
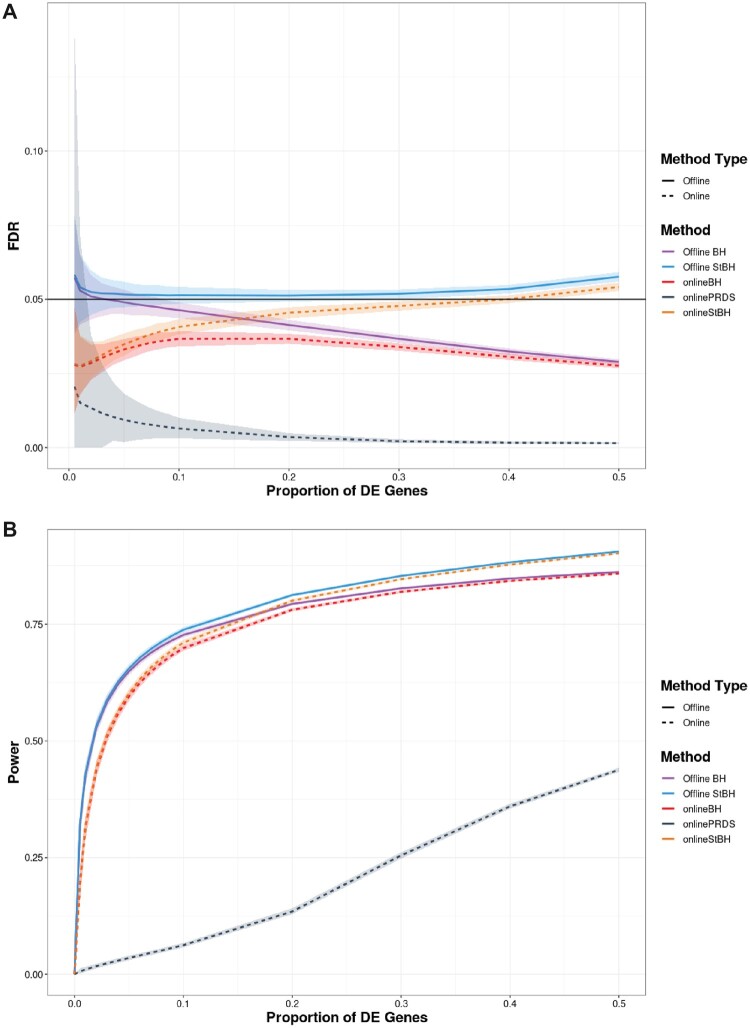
FDR (**A**) and average power (**B**) versus proportion of DE genes (*π*) comparing online and repeated offline algorithms applied to simulated RNAseq count matrices, each with 10 000 genes. Number of simulated count matrices is set to 50 and α=0.05. Simulated log fold-change set to 1.5. Shaded ribbons represent empirical 95% confidence bounds. *π* = 0 is not shown to enhance clarity in visualization, but please refer to Supplementary Figure S1 in the Supplementary Appendix to see results for *π* = 0

We observe that when the repeated offline approaches are used, the standard deviation of FDP is highly variable at low *π* (e.g. π≤0.1), exceeding the pre-specified significance threshold (Fig. 3A). At higher *π*, FDR control is achieved by offline BH. Offline StBH on average slightly inflates the FDR above the threshold with a similarly high extent of variability at low *π* as its BH counterpart. onlineBH and onlinePRDS control FDR well below the significance threshold throughout, with onlinePRDS exhibiting more conservatism. onlinePRDS is also highly variable at very low *π*; however, it still controls FDR at the nominal level as expected from its theoretical guarantees. In our simulation, onlineStBH experiences a slight inflation of FDR at higher π≥0.4.

onlineBH and onlineStBH had closer power to that of offline BH and offline StBH as *π* increased (Fig. 3B). However, onlinePRDS was very conservative across the range of *π* tested. In this simulated scenario, all methods had low power at π<0.1. Figure 3 shows that repeated offline methods do not guarantee control of the global FDR in a repeated application whereas online methods do, while still maintaining comparable levels of power for larger values of *π*.

### 3.3 The effect of ordering

The ordering of different families of RNAseq experiments can be crucial for the outcome of online multiple testing, since it can lead to either a loss or gain of power (Javanmard and Montanari, 2018). To increase power, in some scenarios it may be possible for families of experiments to be ordered, using side information, such that those most likely to have a higher proportion of DE genes are tested first to gain alpha-wealth (i.e. larger values of *α_s_*). To illustrate, if the corresponding gene-*p-*value matrices that have more DE genes could be ordered first, then *α_s_* increases and there is more to spend to make rejections (akin to setting the alpha level greater than the nominal level). On the contrary, if the gene-*p-*value matrices that have fewer DE genes are ordered first, then *α_s_* depletes earlier, and there will be less to spend in subsequent experiments. Hence, we sought to compare the performance of repeated offline and online methods in a simulated ordered setting.

We have simulated a ‘best-case’ scenario in which a scientist decides to order families of RNAseq experiments so that those with a higher proportion of DE genes are tested first, where we assume that there is *a priori* justification. We emphasize that in the context of our simulated RNAseq gene-*p-*value matrices, we are not ordering genes, but rather the gene-*p-*value matrices themselves. We represent this scenario by simulating the first 10 gene-*p-*value matrices (termed ‘Super Matrices’) as having a higher proportion *π* of DE genes, followed by 40 gene-*p-*value matrices (termed ‘Regular Matrices’) that have a lower proportion *π* of DE genes, such that the overall proportion *π* of DE genes is one of {0.1,0.2,0.3,0.4,0.5} (Table 4).

**Fig. 4.**
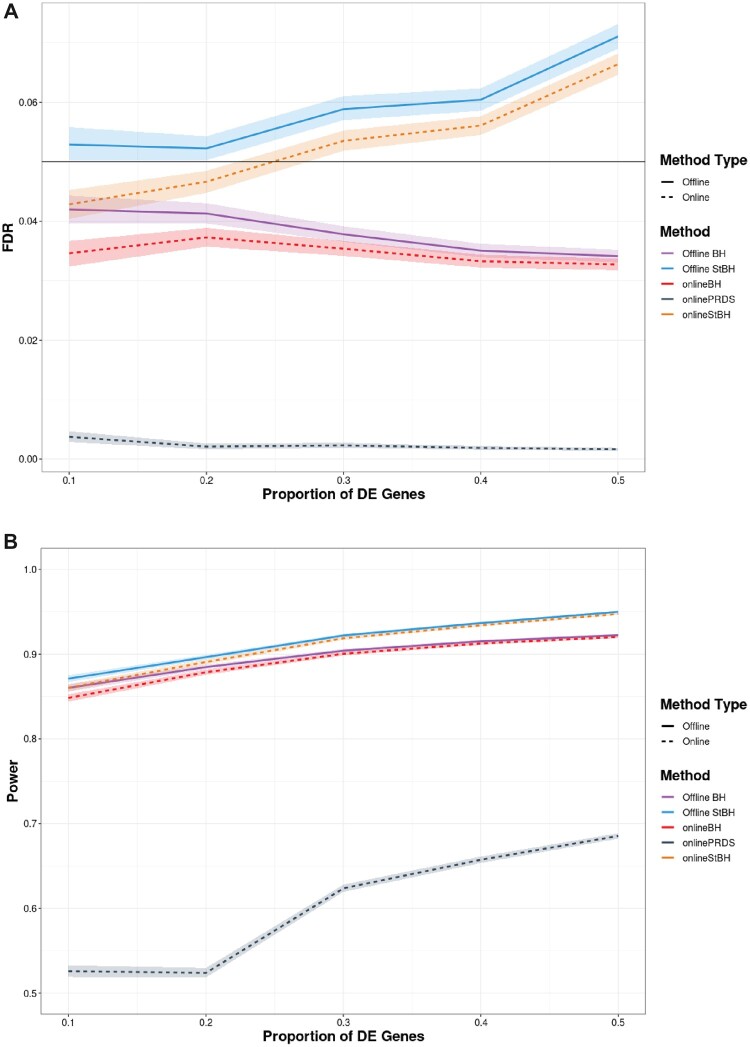
FDR (**A**) and average power (**B**) versus proportion of DE genes (*π*) comparing online and repeated offline algorithms applied to simulated RNAseq count matrices, each with 10 000 genes in an ordered setting such that gene-*p-*value matrices with a higher proportion of differentially expressed genes are tested first. Number of simulated count matrices is set to 50 and α=0.05. Simulated log fold-change set to 1.5. Shaded ribbons represent empirical 95% confidence bounds. *π* = 0 is not shown to enhance clarity in visualization, but please refer to Supplementary Figure S1 in the Supplementary Appendix to see the results for *π* = 0

In this setting, offline StBH does not control FDR at the specified level, across different *π* (Fig. 4A). The ordering of hypothesis tests has benefited offline BH where it now controls the FDR. onlineBH and onlinePRDS still control the FDR at various *π*; however, onlineStBH inflates FDR to comparable levels as π>0.2. We return to this issue in Section 4. The ordering has also benefited all approaches in terms of power. The power achieved by the online algorithms is very close to that achieved by their repeated offline counterparts across all values of *π*.

## 4 Discussion

In this article, we demonstrate that the online FDR control algorithms provide a principled way to guarantee global control of FDR at a nominally specified level in an online paradigm, i.e. families of RNAseq experiments performed over time. We show that repeated application of offline approaches have variable control of FDR over time when the proportion of truly DE genes is love. Furthermore, we observe empirically that online approaches can have comparable power to repeated offline approaches.

The online framework is meant to provide global long-term FDR control. Existing classical offline approaches seek to control for multiplicity across a single family of RNAseq experiments. The online paradigm importantly extends such control to allow for future experiments for which the scientist does not yet have data. In practice, deciding which settings to apply the online framework remains an open question. However, this is part of a broader question of deciding when to apply multiple hypothesis testing in general (Bender and Lange, 2001). Given that our study is the first to apply online multiple hypothesis testing to RNAseq data, we foresee future work continuing to explore the implications of applying this methodology in practice.

Using three real RNAseq gene-*p-*value matrices that we construed as a use case of families of RNAseq experiments arriving over time, we applied the online FDR control methodologies. Although our real-world data were limited in that we could not assess ‘ground truth’, we see that the online methodologies are broadly more conservative than their offline counterparts, which is likely driven by the theoretical long-term FDR control conferred. We also observed that the online methodologies had some face validity in identifying genes related to B-cell activation following anti-PD1 treatment in the experiments filtered out for unique genes. However, we acknowledge one limitation of this analysis in that given the data we used, many of the biologically relevant genes were overlapping in all three experiments, so the filtered non-overlapping genes may have been noise to the true underlying biological mechanisms. Methodologically, an extended framework to allow repeatedly testing the same hypotheses across (families of) experiments remains an important open question.

It is important to note that it was difficult to procure real-world data that exactly fit the use case we described earlier with families of RNAseq experiments for different, yet related treatment conditions. However, given the data, we show that online methodologies successfully recapitulate known biology, and the discovery of novel biology was considered beyond the scope of this study. As high-throughput transcriptome profiling following compound screens becomes more prevalent (Ye *et al.*, 2018), it will be easier to obtain the necessary data to further explore this context. Growing data repositories such as the Gene Expression Omnibus (GEO) are beginning to facilitate budding considerations of global online FDR control use cases across similar RNAseq gene-*p-*value matrices.

Notably, as shown in the simulation studies, as the number of families of RNAseq experiments increase, the online methods are able to recapitulate a higher proportion of genes as detected by more conventional offline methods, which suggests that while the online approaches may have lower statistical power in the short-term, in the long-term, they may have comparable power to repeated offline methods. In our unordered simulation setting, we observed that the online algorithms successfully controlled FDR across a wide range of *π*. Is notable that particularly at low proportions of DE genes, the repeated offline approaches did not maintain control of FDR. The range of *π* that we test may be relevant for the range of number of DE genes that may be detected in practice (Corley *et al.*, 2017). onlineStBH had slight inflation of FDR, but that was at a very high *π* of 0.5 in which the distribution of DE and non-DE genes in an RNAseq experiment is symmetrical, which is likely very rare in practice. The slight inflation of FDR could have also resulted from the subtle dependency introduced in our simulation methods. The *compcodeR* package draws *μ* and ϕ estimates for read count generation from the same underlying real-world data distribution, so it is possible that, although we have simulated the RNAseq count matrices independently to the best of our ability, there still exists some degree of dependency amongst the *p*-values.

In general, our results show onlinePRDS was noticeably more conservative than onlineBH and onlineStBH; it guarantees FDR control when *p-*values are dependent within each family of RNAseq experiments but at the cost of a substantially lower power than any of the other algorithms. However, the fact that the FDR was still controlled when using onlineBH and onlineStBH in the simulations with subtle dependencies amongst *p-*values is reassuring in terms of demonstrating the robustness of these algorithms in departures from independence. In real RNAseq gene-*p-*value matrices, it is likely that DE gene *p-*values are dependent (Subramanian *et al.*, 2005). Future work should investigate the performance of online FDR control methods within varying dependency structures. Subramanian *et al.* (2005) also proposed evaluating sequencing data at the level of gene sets since single-gene analysis may miss important effects on pathways. Thus, one other potential future research direction is to apply online control methods to gene set analysis *p-*values.

We also simulated an ordered setting, since in practice, scientists may be able to order families of experiments in such a way that seeks to optimizes their power and reflects their *a priori* knowledge. We show in our simulations that such ordering increases the power of online methods, but appears to inflate the FDR of onlineStBH at higher values of *π*. In our application of online methods to our real-world data, we did not explore *a priori* ordering of families of RNAseq experiments as biological rationale was not the emphasis. However, it is possible that if we were to test Experiment 2 after both Experiments 1 and 3 (as it appeared inherently less powerful) for some valid biological justification, we may achieve better overall power across the three experiments together in an online framework. Future studies could investigate ordering retrospectively (e.g. in a database such as GEO) or prospectively (e.g. making decisions how to order gene-*p-*value matrices arriving over time). We reiterate that the purpose of our simulation was to explore how much more powerful online methodologies could be if families of experiments with a higher proportion of truly DE genes were tested first. In addition, while we may not expect such a high proportion of DE genes for this to be an issue, nevertheless we caution the use of onlineStBH in an ordered setting as it might ‘greedily’ increase statistical power at the expense of FDR.

Additional future directions of exploring global FDR control across multiple families of RNAseq experiments can explore and benchmark how varying gene-*p*-value matrix size, the *γ_i_* sequence, the number of samples, and using different gene filtering methods or differential expression methods would affect performance of online algorithms. Using other FDR control methods (besides BH and Storey-BH) interpolated in the online manner as we have described is also possible.

### Author contributions

L.L. and D.S.R. conceived the study, L.L. and M.H. obtained the data, L.L. conducted the study, L.L. analysed the results. L.L, M.H. and D.S.R wrote and reviewed the manuscript.

## Funding

This work was supported by the UK Medical Research Council (MC_UU_00002/14 to D.S.R.), the Biometrika Trust (to D.S.R.) and the NIHR Cambridge Biomedical Research Centre (BRC1215-20014 to D.S.R.). The views expressed in this publication are those of the authors and not necessarily those of the NHS, the National Institute for Health Research or the Department of Health and Social Care (DHSC). For the purpose of open access, the author has applied a Creative Commons Attribution (CC BY) license to any Author Accepted Manuscript version arising.

*Conflict of Interest*: At the time of submission, Lathan Liou was an employee at Merck Research Laboratories. Milena Hornburg is a current employee at Merck Research Laboratories.

## Supplementary Material

btac718_Supplementary_Data

## Data Availability

Code used for the simulation studies can be found at https://github.com/latlio/onlinefdr_rnaseq_simulation. The real-world RNAseq data underlying this article will be shared on reasonable request by the corresponding author.
