# Supplementary material for: Global FDR control across multiple RNAseq experiments
Source: Bioinformatics. Author manuscript; Available in PMC 2023 Jan 3. (PMC9805573; doi:10.1093/bioinformatics/btac718)
Supplement: Supplementary File [file EMS156781-supplement-Supplementary_File.pdf]

Global FDR control across multiple RNAseq experiments

Lathan Liou[a], Milena Hornburg[a], and David S. Robertson[b]

[a]Merck & Co., Inc., Kenilworth, NJ, USA
[b]MRC Biostatistics Unit, School of Clinical Medicine, University of Cambridge, UK

November 3, 2022

1 Supplementary Appendix

1.1 Acronyms

**Acronym**	**Meaning**
BH	Benjamini-Hochberg
DE	Differentially Expressed
FDR	False Discovery Rate
FDP	False Discovery Proportion
FWER	Family-wise Error Rate
PD-1	Programmed cell death protein 1
onlinePRDS	Online batch FDR control under positive dependence
StBH	Storey-BH

1.2 Details of Methods

In both the real-world application and the simulation study, we set the γ_i sequences to $\{0.5, 0.5, \ldots, 0\}$ for onlineBH and onlinePRDS as recommended by Zrnic et al. ([Zrn+21]). We used the default $\lambda = 0.5$ for onlineStBH. We use the default γ_i sequence for onlinePRDS.

For our simulation setup, we relied on previous literature to decide RNAseq synthetic data settings. We selected 10,000 genes, 5 samples, and our fold change value of 1.5 following previous RNAseq simulation literature ([Li+19], [Lai17], [YFB17], [BL16], [SD13]). Low sample size has been demonstrated to adversely affect FDR control of differential expression analysis in general ([SD13]). We selected our sequence of π following the online FDR literature ([Zrn+21], [Rob+19]), although in practice, we don't expect the the proportion of truly DE genes to be as high as 0.5. We also set the proportion of DE genes regulated in either the up direction or down direction to 0.5, as Soneson et al. showed that when DE genes were not regulated in different directions, the ability to control the FDR was heavily impaired ([SD13]). We simulated the data using *compcodeR*, which is an R package designed as a tool to benchmark new approaches on

synthetic RNAseq data ([Son14]). We used *limma-voom* as our differential expression method since it is a widely-used method that generates reasonable results ([SD13]).

In the limma-voom workflow, we excluded genes using the *edgeR* method, which is described by Chen et al. (2016) [CLS16]. Removing low count genes may allow for more genes to be declared DE and reduce the computational expense of subsequent analysis. We also use the trimmed mean of M values (TMM) method as described by Robinson and Oshlack [RO10] as our count normalization method.

All code used for this analysis can be found at: `https://github.com/latlio/onlinefdr_rnaseq_simulation`

1.3 Supplementary Tables and Figures

**Experiment**	**Offline BH Genes**	**Offline BH FC (SD)**	**onlineBH Genes**	**onlineBH FC (SD)**	**% Recapitulated**
1	CD27, CD274, CD8A, CMKLR1, CXCL9, CXCR6, HLA-E, IDO1, LAG3, NKG7, PDCD1LG2, PSMB10, STAT1	0.67 (0.38)	CD27, CD274, CD8A, CXCL9, CXCR6, HLA-E, IDO1, LAG3, NKG7, PDCD1LG2, PSMB10, STAT1	0.71 (0.38)	92 (12/13)
2	CD27, CXCR6, HLA-E, LAG3, NKG7, PDCD1LG2, PSMB10, TIGIT	0.72 (0.42)	HLAE, LAG3, NKG7, PSMB10	0.65 (0.33)	50 (4/8)
3	CD27, CD274, CD8A, CMKLR1, CXCR6, HLA-E, IDO1, LAG3, NKG7, PDCD1LG2, PSMB10, STAT1, TIGIT	0.85 (0.48)	CD27, CMKLR1, HLA-E, LAG3, NKG7, PDCD1LG2, PSMB10, TIGIT	0.92 (0.50)	62 (8/13)

Table S 1: Recapitulation of anti-PD1 human gene expression profile as described in Cristescu et al. comparing offline BH and onlineBH methods

Experiment	Offline StBH Genes	Offline StBH FC (SD)	onlineStBH Genes	onlineStBH FC (SD)	% Recapitulated
1	CD27, CD274, CD8A, CMKLR1, CXCL9, CXCR6, HLA-E, IDO1, LAG3, NKG7, PDCD1LG2, PSMB10, STAT1	0.62 (0.38)	CD27, CD274, CD8A, CMKLR1, CXCL9, CXCR6, HLA-E, IDO1, LAG3, NKG7, PDCD1LG2, PSMB10, STAT1	0.64 (0.38)	100 (13/13)
2	CD27, CD8A, CMKLR1, CXCL9, CXCR6, HLA-E, IDO1, LAG3, NKG7, PDCD1LG2, PSMB10, STAT1, TIGIT	0.67 (0.37)	CD27, CD8A, CXCR6, HLA-E, LAG3, NKG7, PDCD1LG2, PSMB10, STAT1, TIGIT	0.72 (0.39)	77 (10/13)
3	CD27, CD274, CD8A, CMKLR1, CXCR6, HLA-E, IDO1, LAG3, NKG7, PDCD1LG2, PSMB10, STAT1, TIGIT	0.78 (0.47)	CD27, CD274, CD8A, CMKLR1, CXCR6, HLA-E, IDO1, LAG3, NKG7, PDCD1LG2, PSMB10, STAT1, TIGIT	0.82 (0.47)	100 (13/13)

Table S 2: Recapitulation of anti-PD1 human gene expression profile as described in Cristescu et al. comparing offline StBH and onlineStBH methods

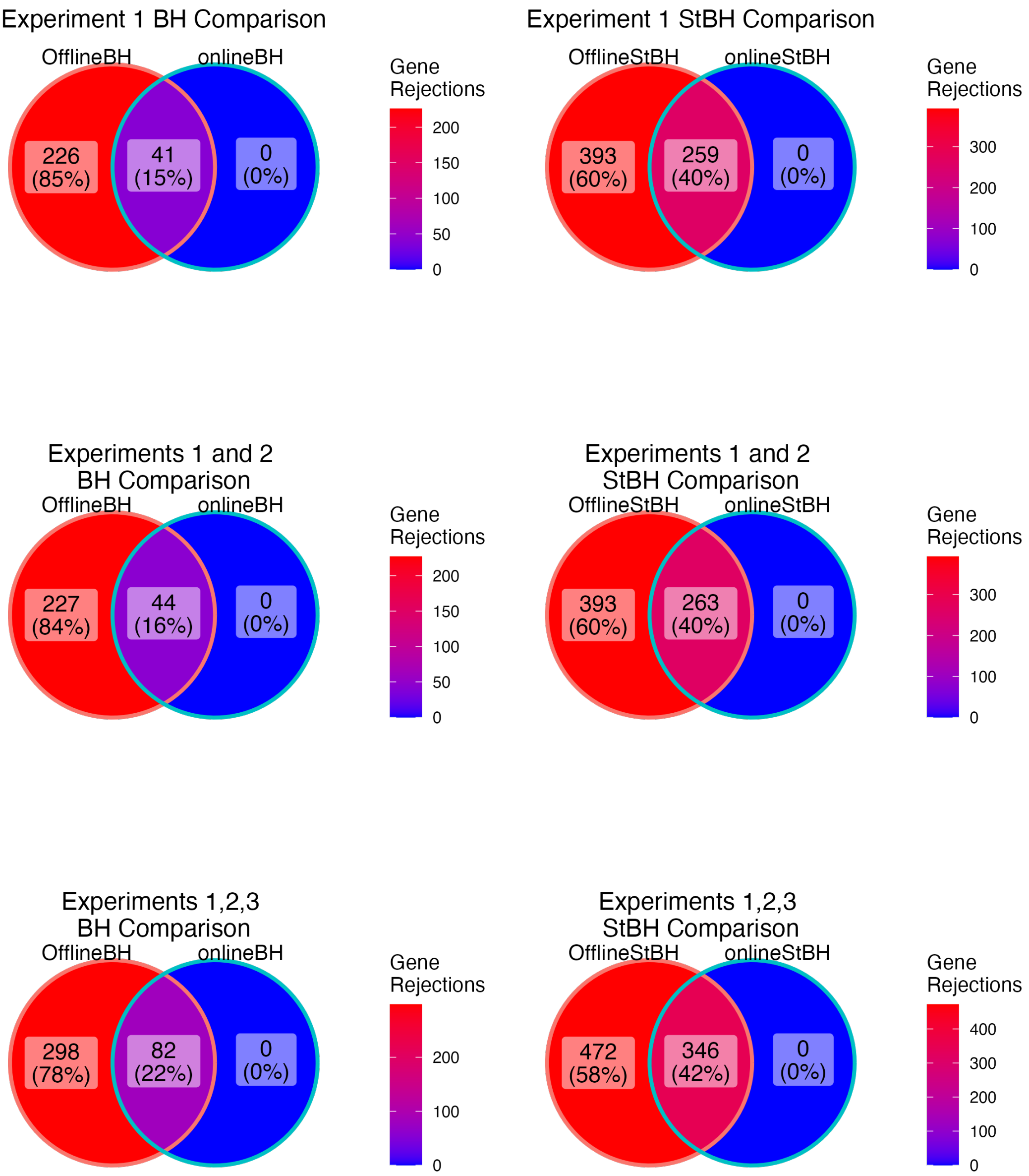

Figure S 1: Overlap in unique filtered genes declared differentially expressed between the repeated offline and online approaches. Numbers of unique differentially expressed genes are summed across experiments 1 and 2, and across experiments 1, 2, and 3. $\alpha = 0.05$

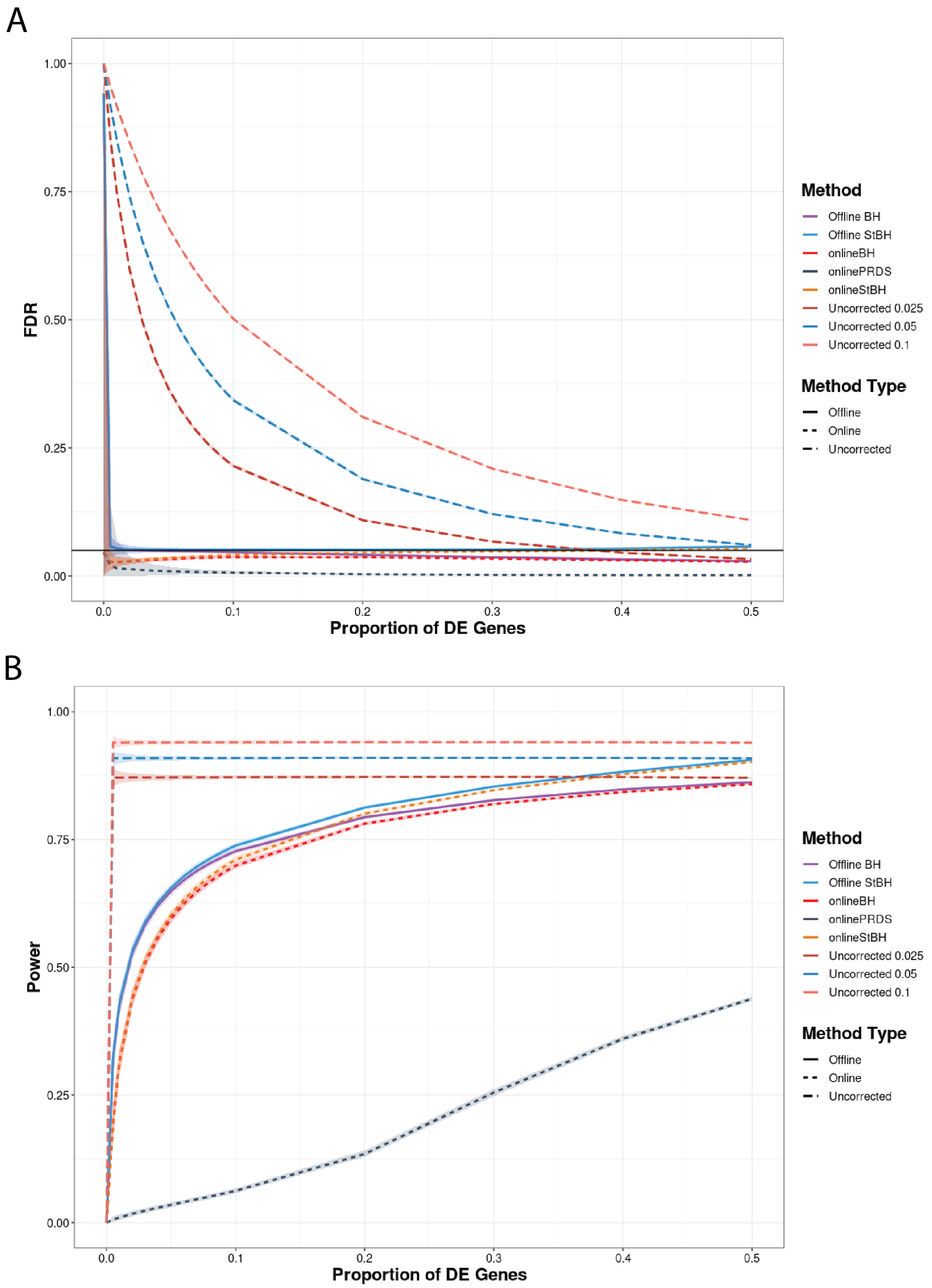

Figure S 2: FDR (A) and average power (B) versus proportion of DE genes (π) comparing offline, pooled offline, and online Batch algorithms for a count matrix with 10000 rows. The number of families of experiments is set to 50, and $\alpha = 0.05$. Simulated log fold-change set to 1.5. Shaded ribbons represent empirical 95% confidence bounds. Showing $\pi = 0$ for completeness.

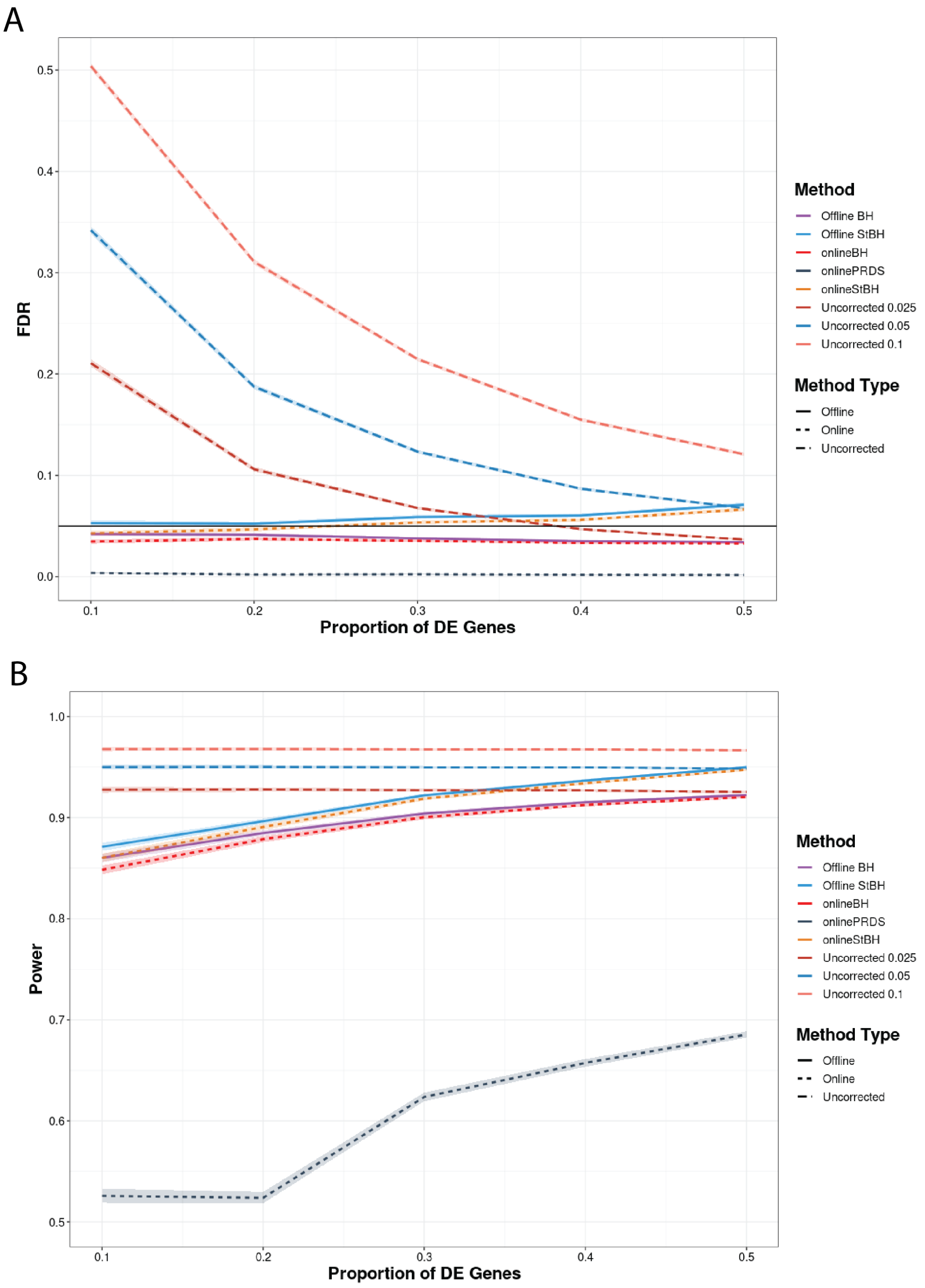

Figure S 3: FDR (A) and average power (B) versus probability of non-null hypotheses π comparing offline and online algorithms for a count matrix with 10000 rows in an ordered setting such that families of experiments with a higher proportion of differentially expressed genes are tested first. Number offamilies of experiments is set to 50 and $\alpha = 0.05$. Simulated log fold-change set to 1.5. Shaded ribbons represent empirical 95% confidence bounds

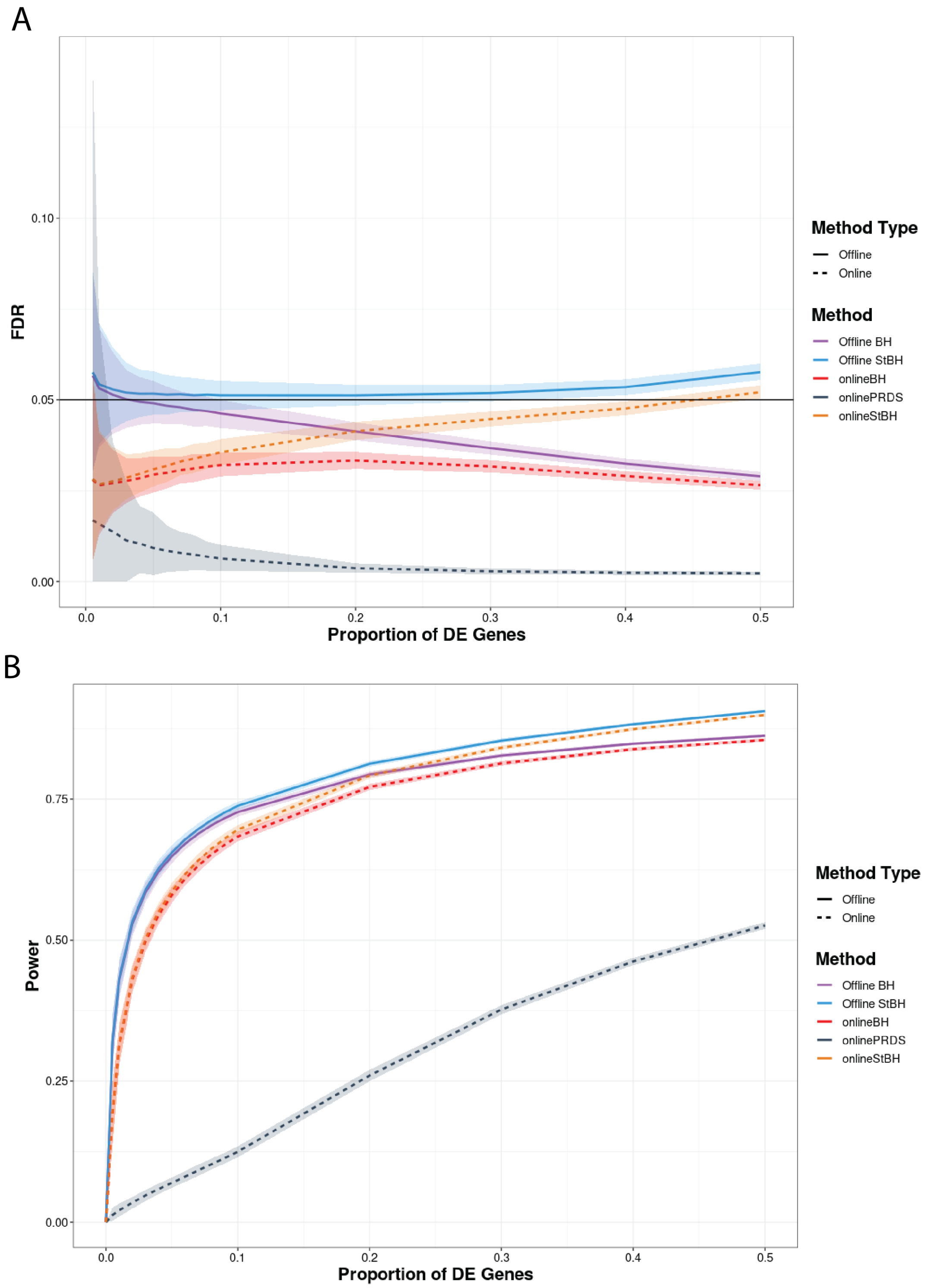

Figure S 4: FDR (A) and average power (B) versus proportion of DE genes (π) comparing offline and online algorithms for a count matrix with 10000 rows. Number of families of experiments is set to 25 and $\alpha = 0.05$. Simulated log fold-change set to 1.5. Shaded ribbons represent empirical 95% confidence bounds. Showing $\pi = 0$ for completeness.

References

[RO10] Mark D Robinson and Alicia Oshlack. “A scaling normalization method for differential expression analysis of RNA-seq data”. eng. In: *Genome biology* 11.3 (2010). Edition: 2010/03/02 Publisher: BioMed Central, R25–R25. ISSN: 1474-760X. DOI: 10.1186/gb-2010-11-3-r25. URL: https://pubmed.ncbi.nlm.nih.gov/20196867.

[SD13] Charlotte Soneson and Mauro Delorenzi. “A comparison of methods for differential expression analysis of RNA-seq data”. In: *BMC Bioinformatics* 14.1 (Mar. 2013), p. 91. ISSN: 1471-2105. DOI: 10.1186/1471-2105-14-91. URL: https://doi.org/10.1186/1471-2105-14-91.

[Son14] Charlotte Soneson. “compcodeR–an R package for benchmarking differential expression methods for RNA-seq data.” eng. In: *Bioinformatics (Oxford, England)* 30.17 (Sept. 2014). Place: England, pp. 2517–2518. ISSN: 1367-4811 1367-4803. DOI: 10.1093/bioinformatics/btu324.

[BL16] Ran Bi and Peng Liu. “Sample size calculation while controlling false discovery rate for differential expression analysis with RNA-sequencing experiments”. In: *BMC Bioinformatics* 17.1 (Mar. 2016), p. 146. ISSN: 1471-2105. DOI: 10.1186/s12859-016-0994-9. URL: https://doi.org/10.1186/s12859-016-0994-9.

[CLS16] Yunshun Chen, Aaron T L Lun, and Gordon K Smyth. “From reads to genes to pathways: differential expression analysis of RNA-Seq experiments using Rsubread and the edgeR quasi-likelihood pipeline”. eng. In: *F1000Research* 5 (June 2016). Publisher: F1000Research, pp. 1438–1438. ISSN: 2046-1402. DOI: 10.12688/f1000research.8987.2. URL: https://pubmed.ncbi.nlm.nih.gov/27508061.

[Lai17] Yinglei Lai. “A statistical method for the conservative adjustment of false discovery rate (q-value)”. In: *BMC Bioinformatics* 18.3 (Mar. 2017), p. 69. ISSN: 1471-2105. DOI: 10.1186/s12859-017-1474-6. URL: https://doi.org/10.1186/s12859-017-1474-6.

[YFB17] Lianbo Yu, Soledad Fernandez, and Guy Brock. “Power analysis for RNA-Seq differential expression studies”. In: *BMC Bioinformatics* 18.1 (May 2017), p. 234. ISSN: 1471-2105. DOI: 10.1186/s12859-017-1648-2. URL: https://doi.org/10.1186/s12859-017-1648-2.

[Li+19] Xiaohong Li et al. “Sample size calculations for the differential expression analysis of RNA-seq data using a negative binomial regression model.” eng. In: *Statistical applications in genetics and molecular biology* 18.1 (Jan. 2019). Place: Germany, /j/sagmb.2019.18.issue–1/sagmb–2018–0021/sagmb–2018–0021.xml. ISSN: 1544-6115. DOI: 10.1515/sagmb-2018-0021.

[Rob+19] David S Robertson et al. “onlineFDR: an R package to control the false discovery rate for growing data repositories”. eng. In: *Bioinformatics (Oxford, England)* 35.20 (Oct. 2019). Publisher: Oxford University Press, pp. 4196–4199. ISSN: 1367-4811.

DOI: 10.1093/bioinformatics/btz191. URL: https://pubmed.ncbi.nlm.nih.gov/30873526.

[Zrn+21] Tijana Zrnic et al. "The Power of Batching in Multiple Hypothesis Testing". In: *arXiv preprint arXiv:1910.04968* (Mar. 2021).
